# Dynamic Xenon-Enhanced Spectral CT Imaging to Evaluate Regional Lung Function in an In Vivo Rabbit Model

**DOI:** 10.3390/diagnostics16203396

**Published:** 2026-10-19

**Authors:** Emma Verelst, Sam Bayat, Sylvia Verbanck, Gert Van Gompel, Johan de Mey, Nico Buls

**Affiliations:** 1 Department of Radiology, Research Centre For Digital Medicine (REDM), Universitair Ziekenhuis Brussel (UZ Brussel), Vrije Universiteit Brussel (VUB), Laarbeeklaan 101, 1090 Brussels, Belgium; 2 Inserm UA07 STROBE Laboratory, Université Grenoble Alpes, Rue De La Piscine 2280, 38400 Grenobles, France; 3 Department of Pneumology, Universitair Ziekenhuis Brussel (UZ Brussel), Vrije Universiteit Brussel (VUB), Laarbeeklaan 101, 1090 Brussels, Belgium

**Keywords:** xenon, dual-energy CT, pulmonary functional imaging, in vivo imaging, animal study

## Abstract

**Background/Objectives**: Pulmonary diseases exhibit spatially heterogeneous functional and structural abnormalities that are inadequately represented by conventional lung function testing. This pilot study investigates dynamic xenon-enhanced dual-energy CT (Xe-DECT) for a multiparametric assessment of lung ventilation and tissue. **Methods**: Four mechanically ventilated rabbits were scanned during washout of 70% xenon in oxygen using multibreath imaging with a clinical DECT both before (baseline) and after methacholine administration (post-MCh). Tracheal Xe concentration (*C_Xe_*) and flow were monitored using mass spectrometry and pneumotachometry to estimate specific ventilation (${s\dot{V}}_{\mathrm{ms}}$) and end-expiratory lung volume (EELV_ms_). DECT-derived xenon concentration and tissue density images (mg/mL) were obtained by material decomposition, yielding ${s\dot{V}}_{\mathrm{DECT}}$ and EELV_DECT_, respectively. Agreement with mass spectrometry and differences between baseline and post-MCh were assessed. **Results**: The baseline ${s\dot{V}}_{\mathrm{DECT}}$ and EELV_DECT_ agreed with the mass spectrometry, with mean differences of 0.6 min^−1^ [−0.2 min^−1^, 1.4 min^−1^] and 0.6 mL [−6.7 mL, 7.9 mL], respectively. Following MCh administration, the mean differences increased to −2.9 min^−1^ [−4.1 min^−1^, −1.6 min^−1^] for $s\dot{V}$ and 1.3 mL [−15.3 mL, 12.8 mL] for EELV. Post-MCh, ${s\dot{V}}_{\mathrm{DECT}}$ decreased from 8.6 ± 1.6 min^−1^ to 3.7 ± 0.9 min^−1^ (*p* = 0.034) and EELV_DECT_ from 29.3 ± 1.0 mL to 27.0 ± 1.3 mL (*p* = 0.036), whereas ${s\dot{V}}_{\mathrm{ms}}$ (*p* = 0.308) and EELV_ms_ (*p* = 0.653) showed no substantial changes. Regional analysis revealed that ${s\dot{V}}_{\mathrm{DECT}}$ in well-ventilated regions matched tracheal ${s\dot{V}}_{\mathrm{ms}}$. **Conclusions:** Dynamic Xe-DECT imaging enabled spatial and quantitative assessments of specific ventilation and tissue densities in an in vivo rabbit model. This technique allowed detection of focal alterations that remained undetected by tracheal measurements.

## 1. Introduction

Virtually all pulmonary diseases exhibit spatial heterogeneity in structural and functional abnormalities [1]. While structural heterogeneity is routinely assessed with thoracic computed tomography (CT) or radiography, pulmonary function testing in the pneumology department usually involves spirometry as a primary measure [2]. Although specific changes in spirometric indices reflect specific respiratory pathologies, spirometry merely provides a global measure of lung function. As a result, early-stage lung disease, often reflected by subtle heterogeneous changes, can remain undetected, and healthy lung regions may compensate for heterogeneous functional impairment elsewhere [3].

Various pulmonary functional imaging techniques have been investigated to assess regional lung function [4]. Within the nuclear imaging field, ^99m^Tc-Technegas single-photon emission CT (^99m^Tc-SPECT) ventilation–perfusion (V/Q) imaging is clinically used to diagnose pulmonary embolisms [5], while Galligas-68 positron emission tomography (^68^Ga-PET) ventilation imaging remains confined to specialized centers due to infrastructural requirements [6]. Magnetic resonance imaging (MRI) enables assessment of alveolar-capillary gas exchange or ventilation using hyperpolarized gases, such as ^129^xenon or ^3^helium, or through oxygen-enhanced approaches [7]. More recently, dynamic MRI techniques have been proposed to characterize regional respiratory dynamics [8]. Despite their functional capabilities, nuclear imaging and MRI generally provide limited spatial resolution, while simultaneous assessment of detailed structural abnormalities requires additional imaging, increasing patient and radiation burden.

Computed tomography (CT) provides high-resolution anatomical information but lacks intrinsic functional information [9]. Consequently, computational methods have been developed to estimate regional ventilation from changes in lung volume or attenuation between distinct respiratory phases, commonly referred to as CT-based ventilation imaging (CTVI) [10]. Nevertheless, CTVI remains an indirect method and is highly dependent on image registration accuracy. A more direct CT-based method to image ventilation involves the use of a gaseous contrast agent such as xenon (Z = 54), i.e., Xe-CT [11,12]. However, Xe-CT is acquired at a single tube potential (kVp), limiting xenon–tissue differentiation and the simultaneous assessment of (non-enhanced) lung structure [13].

Contrast-enhanced spectral CT imaging, including dual-energy CT (DECT) or photon-counting CT (PCCT), leverages the energy dependence of tissue and material-specific X-ray attenuation by acquiring information at two (or more) energy levels [14,15]. In combination with inhaled xenon gas, spectral CT enables differentiation of xenon from surrounding lung tissue using material decomposition algorithms [16]. The resulting material-specific images allow mapping and quantification of xenon distribution and lung density, reflecting functional and structural heterogeneity, respectively [17]. Static xenon-enhanced DECT (Xe-DECT) has been previously investigated as a quantitative imaging technique for assessing ventilation heterogeneity patterns in patients suffering from respiratory diseases [18,19,20]. However, spectral imaging in these studies was restricted to a single-breath-hold acquisition, where xenon was measured, but no lung tissue density distributions were investigated.

A comprehensive assessment of pulmonary function requires temporal information to characterize regional gas kinetics as ventilation heterogeneity manifests through local alterations in respiratory dynamics during active breathing [21]. In addition, simultaneous anatomical assessment may facilitate direct correlation between functional abnormalities and underlying structural heterogeneity of the lung tissue [22]. In our previous work, we identified functionally impaired areas as ventilation defects on a static xenon concentration image and then used time-resolved xenon signals to scrutinize the ventilation dynamics in these specific areas [23]. In this paper, we build further upon this approach, considering the dynamics of ventilatory heterogeneity over the entire lung and investigating the feasibility of our Xe-DECT imaging approach to simultaneously map and quantify structural heterogeneity. This is done in healthy and pathological in vivo rabbit lungs using physiologically relevant imaging metrics and by comparing these against independent measurements by mass spectrometry at the trachea.

## 2. Materials and Methods

### 2.1. Animal Model

All procedures and set-ups were approved by the ethical committee for experiments of the host institution, conducted in accordance with EU Regulation 2019/1010, and performed as previously described [23,24]. No unexpected adverse events occurred during the experiments. Four male New Zealand White rabbits (average weight: 2.6 kg ± 0.2 kg; Charles River, L’Arbresle, France) were anesthetized and tracheostomized for mechanical ventilation using a custom-built gas administration system. To obtain a pathological ventilation condition, bronchoconstriction was induced by administering 40 mg/mL methacholine (MCh).

### 2.2. Ventilation Set-Up

#### 2.2.1. Gas Administration

Figure 1 represents a schematic of the ventilation set-up used for gas administration and monitoring at the trachea. A continuous adjustable gas supply was delivered through the inspiratory branch. The respiratory rate was set at 35 cycles/min with a tidal volume (TV) of 6–7 mL/kg. A xenon wash-in was initiated using 70% stable xenon in 30% oxygen (O_2_), followed by xenon washout after switching to an air mixture. The xenon washout phase was used for further analysis.

#### 2.2.2. Xenon and Airflow Monitoring at the Trachea

Air, xenon concentration (*C_Xe_* in mg/mL) and O_2_ were continuously monitored using a mass spectrometer (LR6000, Logan Research, Rochester, UK), while flow rate (mL/s) was monitored using a heated Fleisch pneumotachometer (Hans Rudolph, Kansas City, MO, USA); both were sampled at the endotracheal tube outlet. *C_Xe_* and flow rate were used to calculate two lung function metrics: specific ventilation ($s\dot{V}$ in min^−1^) and end-expiratory lung volume (EELV in mL). $s\dot{V\;}$ describes ventilation efficiency by modeling xenon gas kinetics and was quantified as the time constant (min^−1^) of a normalized mono-exponential model [25] using the time-resolved mass spectrometry data (${s\dot{V}}_{\mathrm{ms}}$) (Equation (1)):

(1) $$C_{\mathrm{Xe}}(t)={C_{\mathrm{Xe},\;t0}\times e}^{-s\dot{V}\cdot t}$$

where *C_Xe_*_,*t*0_ represents the xenon concentration measured by mass spectrometry at the start (*t*_0_) of the washout.

EELV by mass spectrometry (EELV_ms_) was computed using a mass-balance approach, which considers that the rabbit is equilibrated with xenon at the onset of the washout phase (*C_Xe_*_,*t*0_) (Figure 1c), breathes out a mean xenon concentration (*C*_*Xe*,*exp*_) over the tidal volume (TV) at each subsequent washout expiration, and remains at a final xenon concentration at the end of washout (*C_Xe_*_,*end*_) (Equation (2)). Thus, EELV becomes

(2) $${\mathrm{EELV}}_{\mathrm{ms}}=\frac{\sum_{i=0}^{n}\left(\mathrm{TV}\;\cdot C_{\mathrm{Xe},\exp}\right)\;}{C_{\mathrm{Xe},t0}-C_{\mathrm{Xe},\mathrm{end}}},$$

### 2.3. CT Imaging and Multiparametric Processing

#### 2.3.1. CT-Imaging Protocol and Reconstruction

Scanning was performed before (baseline) and after MCh administration (post-MCh) using a fast kV-switching clinical DECT (GE Revolution, General Electric Healthcare, Chicago, IL, USA) in spiral shuttle mode. Fifteen multi-breath images were acquired, totaling a cumulative CT volumetric dose index (CTDI_vol_) of 71 mGy and a dose length product (DLP) of 85.30 mGy·cm. Further details on scan and reconstruction settings can be found in Table 1.

DECT data were decomposed into xenon and water density images (expressed in mg/mL) using a standard two-material DECT decomposition model that was available on a dedicated clinical dual-energy workstation (AW VolumeShare 7, Release 4.7, General Electric Healthcare, Chicago, IL, USA). In this model, the low- and high-energy attenuation coefficients were modeled as a linear combination of xenon and water [13]. The energy-dependent mass attenuation coefficients for both xenon and water were assumed known, with xenon values obtained from the National Institute of Standards and Technology (NIST) database [26]. The water density images were interpreted as representing the tissue component of the lung parenchyma.

#### 2.3.2. Image Processing

To ensure temporal correspondence between image and ventilation monitoring data, segmented full lung volumes (cm^3^-mL) and the mean xenon density at each imaging time point were aligned with the airflow and xenon concentration traces at the trachea. This synchronization allowed for accurate identification of inhale and exhale imaging time points that were considered for further analysis of xenon and tissue density images, as illustrated in Figure 2.

The inhale xenon density images required correction for the presence of lung tissue by multiplying the xenon density (*D_Xe_*) with the tissue–gas correction factor (*f_cor_*), as previously discussed [23]. This yielded a corrected xenon density (*D′_Xe_*), representing the local xenon concentration within each voxel (Equations (3) and (4)):

*D′_Xe_* = *D_Xe_* ∗ *f_cor_* (3)

where *f_cor_* is expressed as

(4) $$f_{\mathrm{cor}}=\frac{\left(D_{\mathrm{air}}-D_{t}\right)}{\left(D_{l}-D_{t}\right)},$$

where *D_l_*, *D_t_* and *D_air_*, the densities of lung, tissue and air, respectively, were obtained from the tissue density images in mg/mL. *D_l_* was measured at each time point and in each voxel, providing a time series of three-dimensional matrices. *D_t_* was measured in the spinal muscle tissue (assuming a similar density to that of the tissue phase of the lung parenchyma) and *D_air_* in the trachea.

The xenon concentration images (*D′_Xe_*) were registered to the first inspiratory washout image using a B-spline deformable image registration algorithm implemented from SimpleITK (version 2.0.2 in Python) [27]. $s\dot{V}$ was quantified on a voxel-wise level by exponential modeling of the time-resolved and normalized *D′_Xe_* (Equation (5)) [25]:

(5) $${D\prime }_{\mathrm{Xe}}(t)={{D\prime }_{\mathrm{Xe},t0}\times e}^{-s\dot{V}\cdot t}$$

where *D′_Xe_*_,*t*0_ represents the *D′_Xe_* at the start (t_0_) of the washout. Voxels for which exponential fitting failed due to little or no xenon enhancement were assigned an $s\dot{V}$ of zero.

#### 2.3.3. Multiparametric Image Assessment

DECT-based $s\dot{V}$ (${s\dot{V}}_{\mathrm{DECT}}$) was computed as the average time constant of the normalized time-resolved *D′_Xe_* data (Equation (5)), derived from volumetric parenchymal masks of the xenon concentration images, excluding all conducting airways and large blood vessels. In addition, ${s\dot{V}}_{\mathrm{DECT}}$ was also regionally investigated in poorly ${(s\dot{V}}_{\mathrm{poor}})$ and well (${s\dot{V}}_{\mathrm{well}})$ ventilated lung regions. A threshold was selected empirically to provide consistent delineation of ventilation defects across all animals. Poorly ventilated regions were classified as voxels with $s\dot{V}$ lower than 2 standard deviations (SDs) below ${s\dot{V}}_{\mathrm{DECT}}$, whereas all remaining voxels were classified as ${s\dot{V}}_{\mathrm{well}}$.

DECT-based EELV (EELV_DECT_) was computed using end-exhalation imaging time points (Figure 2) as the volume of air inside the parenchyma by multiplying the segmented lung volume with the volume gas fraction (*f_gas_*) (Equation (6)). Similarly to *f_cor_* (Equation (3)), *f_air_* was computed using the voxel-wise *D_l_* and the average *D_t_* and *D_air_* set to 0.001 mg/mL:

(6) $$f_{\mathrm{air}}=\;\frac{\left(D_{l}\;-\;D_{t}\right)}{\left(D_{\mathrm{air}}\;-\;D_{t}\right)},$$

### 2.4. Statistics

Continuous variables were expressed as means, accompanied by their associated SDs or limits of agreement (LoAs).

Agreement between DECT- and mass spectrometer-based $s\dot{V}$ and EELV was assessed separately for each ventilation condition using a Bland–Altman analysis. In addition, DECT and mass spectrometer-based $s\dot{V}$ and EELV were compared between baseline and post-MCh conditions using a paired-samples *t*-test. Prior to the paired-samples *t*-test, data were assessed using a Shapiro–Wilk test. All statistical analyses were performed using Python (version 3.11.8, Python Software Foundation, Wilmington, DE, USA).

## 3. Results

Figure 3 shows the ventilation ($s\dot{V}$) maps and tissue density images. In baseline conditions, relatively homogeneous patterns were observed, with a subtle increase in $s\dot{V}$ and tissue density towards the dorsal lung regions; in these experiments, dorsal lung regions are the dependent regions in the line of gravity. During MCh-induced bronchoconstriction (post-MCh), a marked redistribution of $s\dot{V}$ was detected, consistent with the corresponding heterogeneous tissue density pattern. Certain dorsal areas also showed absent ventilation ($s\dot{V}$ = 0), coinciding with severely constricted areas at a tissue density above approximately 3 mg/mL.

The mean $s\dot{V}$ and EELV values obtained by DECT imaging and mass spectrometry are summarized in Table 2. Figure 4 illustrates the Bland–Altman agreement analyses. At baseline, ${s\dot{V}}_{\mathrm{DECT}}$ and ${s\dot{V}}_{\mathrm{ms}}$ showed a mean difference of 0.6 min^−1^ and 95% LoAs, ranging from −0.2 min^−1^ to 1.4 min^−1^. Following MCh administration, the mean difference increased to −2.9 min^−1^, with 95% LoAs ranging from −4.1 min^−1^ to −1.6 min^−1^. Compared with baseline, the post-MCh ${s\dot{V}}_{\mathrm{DECT}}$ decreased by 4.9 ± 2.3 min^−1^ (*p* = 0.034), whereas ${s\dot{V}}_{\mathrm{ms}}$ decreased by 1.4 ± 2.0 min^−1^ (*p* = 0.308). The regional ${s\dot{V}}_{\mathrm{DECT}}$ revealed a 0.5 ± 0.9 min^−1^ difference between ${s\dot{V}}_{\mathrm{well}}$ and ${s\dot{V}}_{\mathrm{ms}}$ (*p* = 0.351), whereas ${s\dot{V}}_{\mathrm{poor}}$ differed by 6.4 ± 0.8 min^−1^ from ${s\dot{V}}_{\mathrm{ms}}$ (*p* < 0.001).

The baseline EELV_DECT_ and EELV_ms_ showed a mean difference of 0.6 mL and 95% LoAs ranging from −6.7 mL to 7.9 mL that increased to 1.3 mL, with 95% LoAs ranging from −15.3 mL to 12.8 mL following MCh administration. Compared with baseline, the post-MCh EELV_DECT_ decreased by 3.5 ± 6.2 mL (*p* = 0.036), whereas EELV_ms_ decreased by 0.7 ± 6.3 mL (*p* = 0.653).

## 4. Discussion

The lung is a highly complex system in which parenchymal architecture and mechanical properties determine and regulate its function [28]. Building on our prior rabbit study that established a first proof-of-concept of the animal model, the gas administration, the Xe-DECT imaging and reconstruction protocols and subsequent tissue volume correction of individual xenon images, the present pilot expands on this framework by evaluating the feasibility of dynamic Xe-DECT imaging to assess lung function, considering both ventilatory dynamics and tissue heterogeneity on a voxel-wise level over the entire lung. Our results demonstrate the potential of dynamic Xe-DECT scanning for a spatial and quantitative evaluation of local ventilation efficiency, $s\dot{V}$, and lung inflation, aeration and tissue density.

We considered a voxel-wise $s\dot{V}$ representation to assess ventilation efficiency heterogeneity. The ventilation maps revealed dorsal regions of reduced ventilation efficiency following MCh administration, accounting for the overall reduction in the average ${s\dot{V}}_{\mathrm{DECT}}$. These findings are consistent with the results from Bayat S et al. [29], who demonstrated similar patterns of $s\dot{V}$ heterogeneity using xenon-enhanced synchrotron radiation imaging with K-edge subtraction following the administration of a MCh aerosol in a similar in vivo rabbit model that was scanned in an upright position [29]. Notably, despite the difference in animal positioning (supine in our study and upright in Bayat et al.’s [29]), poorly ventilated areas were observed in the same dorsal lung areas. This consistency suggests that the spatial distribution of ventilation defects may be governed predominantly by intrinsic regional airway anatomy and lung mechanics rather than by gravitational effects [30].

EELV was included as a second metric to describe regional lung inflation, aeration and tissue density properties because the rabbits were mechanically ventilated. Under normal (spontaneous) breathing conditions, EELV is equal to the functional residual capacity (FRC), routinely measured using body plethysmography or washout testing in the lung function lab. Pulmonary volumes, and FRC in particular, are a key factor in many restrictive and obstructive lung diseases, such as pulmonary fibrosis and COPD [31]. In the present study, the EELV was measured using tissue density images. In line with the $s\dot{V}$ findings, showing large regions with little ventilation (aeration) after MCh administration, a reduction in EELV_DECT_ was observed. This is likely attributable to airway closure and heterogeneous tissue constriction, as described by Strengell et al. [32]. This was further evidenced by our post-MCh tissue density maps, which demonstrated severely constricted areas in the dorsal lung. In fact, these dorsal regions showed such severe tissue constriction, suggesting complete atelectasis, that it precluded xenon enhancement in these areas, affecting the calculation of specific ventilation. These regions appeared on the ventilation maps with a $s\dot{V}$ of zero and accounted for the low $s\dot{V}$ values observed in the poorly ventilated regions.

The observed baseline agreements between DECT- and mass spectrometry-based metrics support the quantitative capability of dynamic Xe-DECT scanning. Following MCh administration, agreement decreased, likely reflecting foci of functional and structural heterogeneity visible on our ventilation and tissue density maps. However, the DECT-derived $s\dot{V}$ in well-ventilated areas was very similar to $s\dot{V}$ measured by mass spectrometry at the level of the trachea. This illustrates fundamental differences in xenon signal quantification between the two methods. Mass spectrometry samples gas at the airway opening, producing a flow-weighted xenon signal and, hence, poorly representing severely constricted and poorly ventilated regions. In contrast, DECT-derived xenon measurements are volume-weighted, with voxels from all lung regions contributing equally to the average xenon density, irrespective of their degree of ventilation. Consequently, poorly ventilated regions are duly represented in the DECT-derived signal but are underrepresented in the mass spectrometry measurements. Therein lies a potential advantage of DECT when spatially resolving and mapping heterogeneous pulmonary impairment, particularly during the early stages of, or in focal, small airway disease.

Our study has several limitations. We did not consider time-resolved data from the wash-in cycle because mechanical ventilation altered xenon wash-in kinetics and therefore prevented a reliable assessment of the baseline physiological ventilation response. Furthermore, a relatively broad limit of agreement was observed for EELV results. This increased variability is likely attributable to pronounced bronchoconstriction induced by MCh, resulting in substantial regional tissue density changes and making lung volume computation more sensitive to small differences in lung inflation between end-exhale times arising from small inconsistencies in the manual synchronization between ventilation and imaging points. These findings indicate a more optimized methodology might be required to further investigate Xe-DECT’s potential to compute EELV, particularly for acquiring sequential images in pathological conditions. In addition, this study considered an animal model. Differences in lung size, airway anatomy and respiratory mechanics between rabbits and humans may limit the direct translation of these findings to clinical applications. Lastly, given the small sample size (*n* = 4), the statistical analyses were considered exploratory, and the results were interpreted in the context of this pilot study.

The present dynamic Xe-DECT approach holds considerable potential in future clinical applications such as functional lung avoidance therapy [12] or post-operative lung prediction [33]. Nevertheless, several challenges remain before clinical translation. Our imaging protocol in this feasibility study should be optimized towards lower xenon concentrations. Clinical implementation would require the use of xenon concentrations ranging between 30% and 35% to limit its sedative properties [16,34,35]. Although Chon et al. [36] observed increased relative image noise at 30%, their findings also suggested that temporal metrics were less affected by reduced xenon concentrations [36]. Second, repetitive imaging results in a substantial radiation burden, necessitating further protocol optimization to reduce radiation dose while maintaining image quality and quantitative accuracy. Finally, the introduction of photon-counting CT technology may facilitate clinical translation through improved xenon sensitivity, lower radiation dose, and higher spatial resolution [37].

## 5. Conclusions

Dynamic Xe-enhanced spectral CT imaging using a clinical DECT system enabled a simultaneous assessment of regional ventilation efficiency and lung inflation, aeration and tissue density in both healthy and pathological in vivo rabbit lungs. Spatially resolved measurements of $s\dot{V}$ and EELV allowed comparison between structural and functional heterogeneity induced by MCh administration. The findings of the present pilot study support the potential of dynamic Xe-DECT for a more complete lung function assessment on a regional level, potentially to facilitate early detection of pulmonary disease.

## Figures and Tables

**Figure 1:**
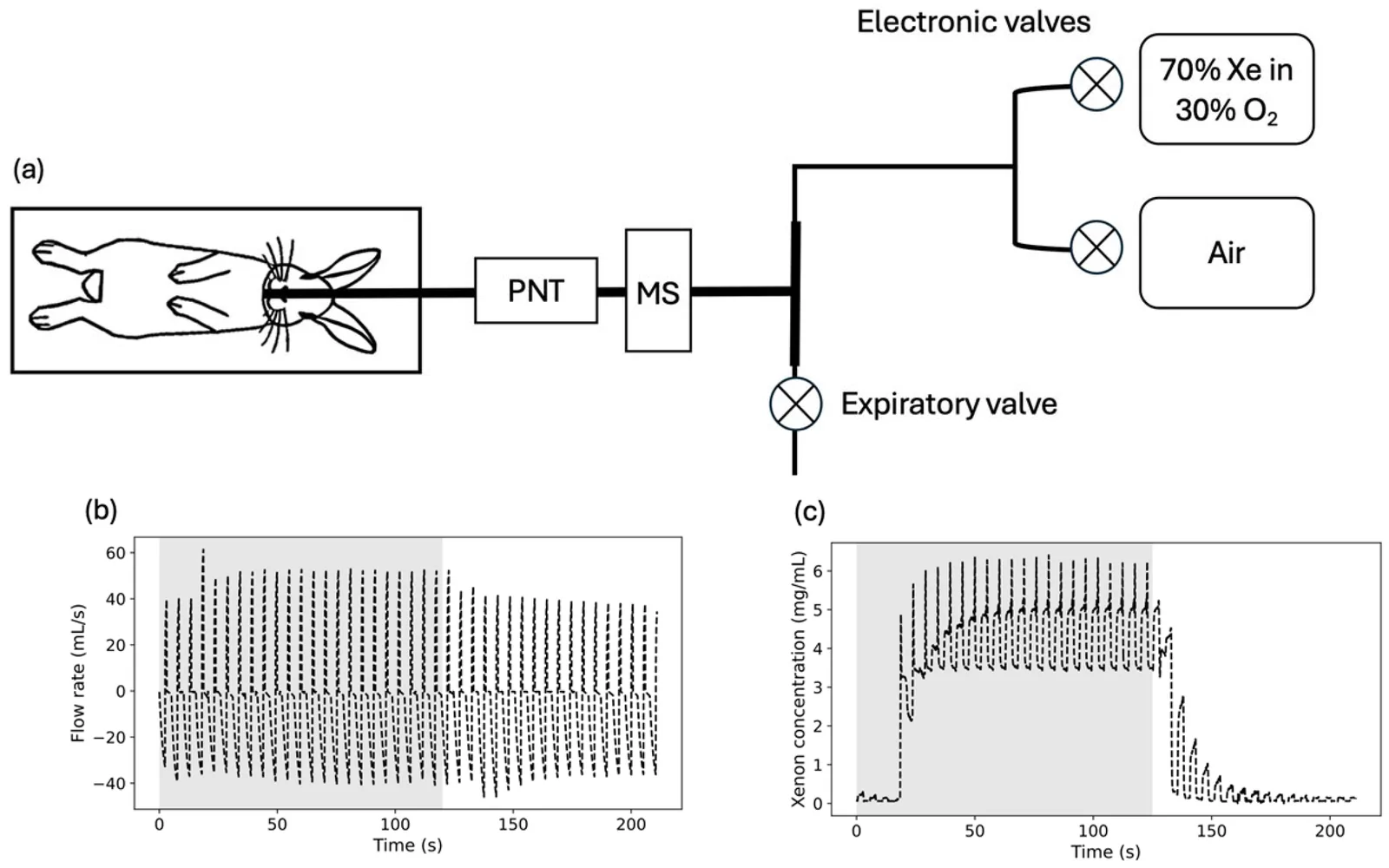
Schematic of the ventilation set-up used for gas administration and xenon and airflow monitoring at the trachea (**a**). A pneumotachometer (PNT) and mass spectrometer (MS) were placed in the endotracheal tube outlet to monitor flow rate (mL/s) (**b**) and xenon (Xe) gas concentration (mg/mL) (**c**), respectively. A xenon wash-in was initiated by administering a mixture of 70% xenon with 30% oxygen, as indicated in grey in panels (**b**,**c**). Xenon washout followed by remotely switching the electronic valves to pure air administration was included for further analyses. The xenon signals in panel (**c**) show pressure overshoots at the end of expiration.

**Figure 2:**
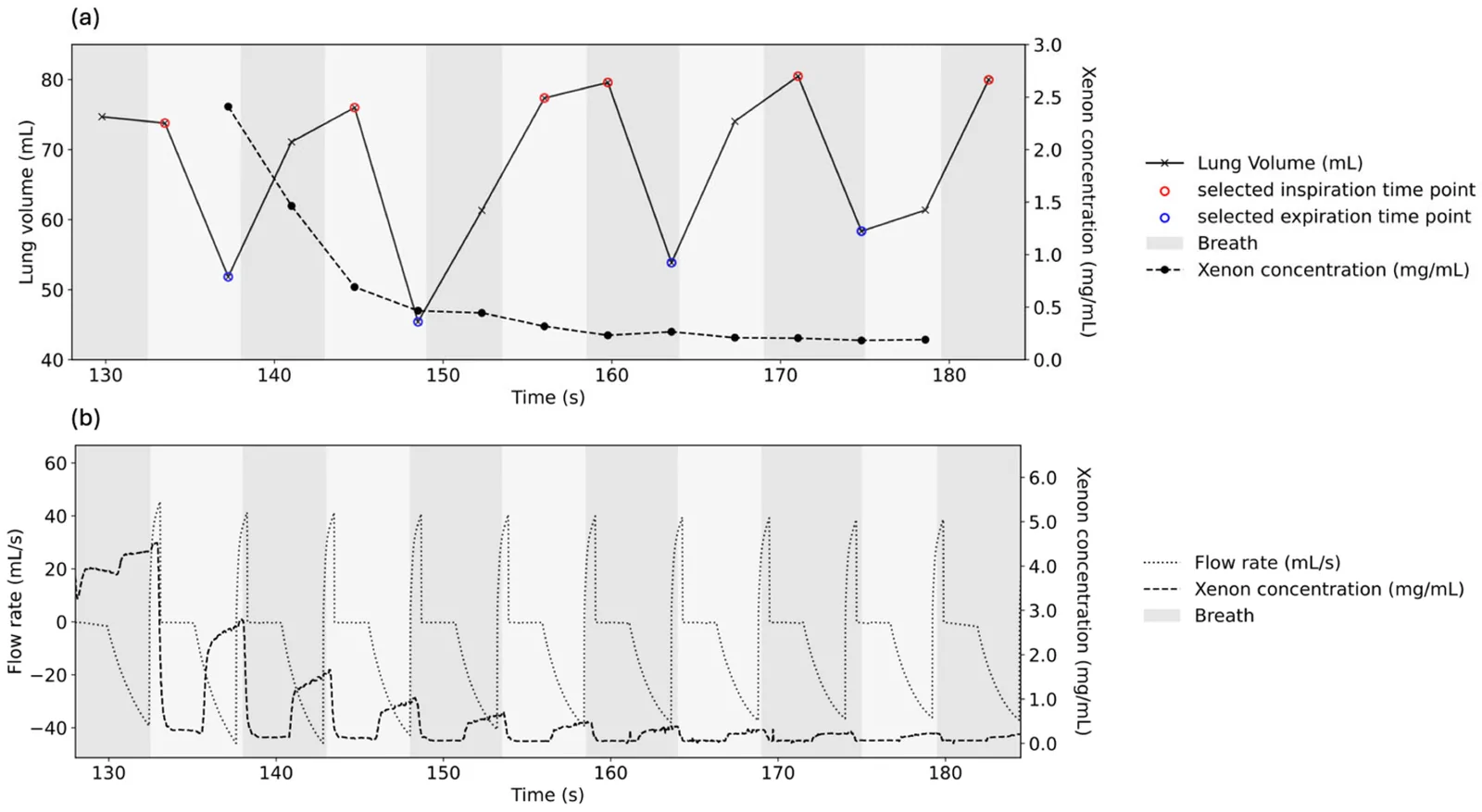
Washout registration between dual-energy CT (DECT)-based lung volumes (mL) and xenon concentrations (mg/mL) (**a**), and flow rate (mL/s) and xenon concentration (mg/mL) traces measured using pneumotachometry and mass spectrometry at the level of the trachea (**b**). The synchronization enabled accurate selection of inhale (red) and exhale (blue) imaging time points for further analysis.

**Figure 3:**
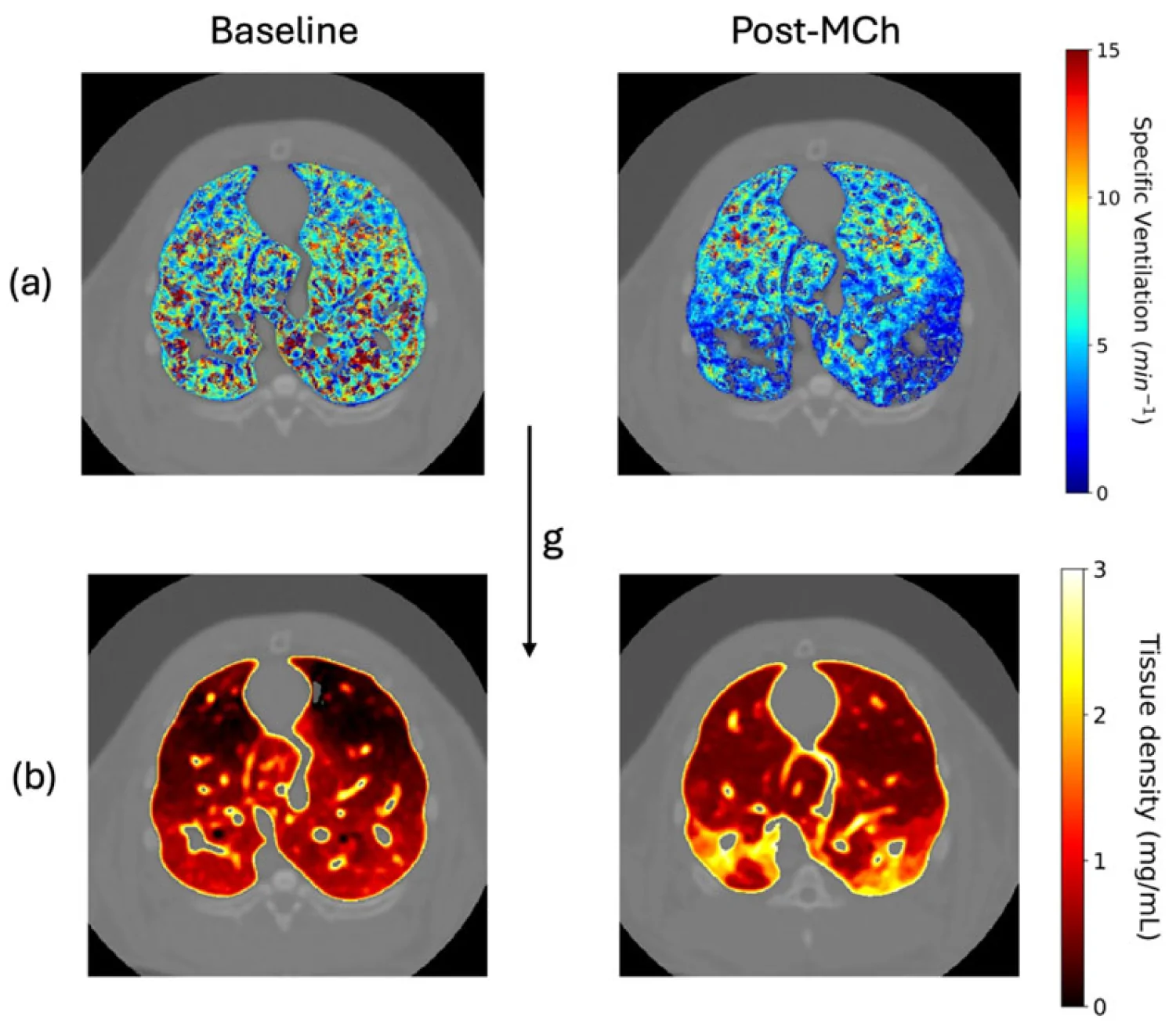
Ventilation maps showing the voxel-wise distribution of specific ventilation (min^−1^) (**a**), and tissue density (mg/mL) images (**b**) in a rabbit scanned in the supine position at baseline (left) and following methacholine (MCh) administration (post-MCh, right). At baseline, a gravitational gradient is observed (arrow). Post-MCh, specific ventilation is redistributed, consistent with bronchoconstriction-induced changes in regional tissue density.

**Figure 4:**
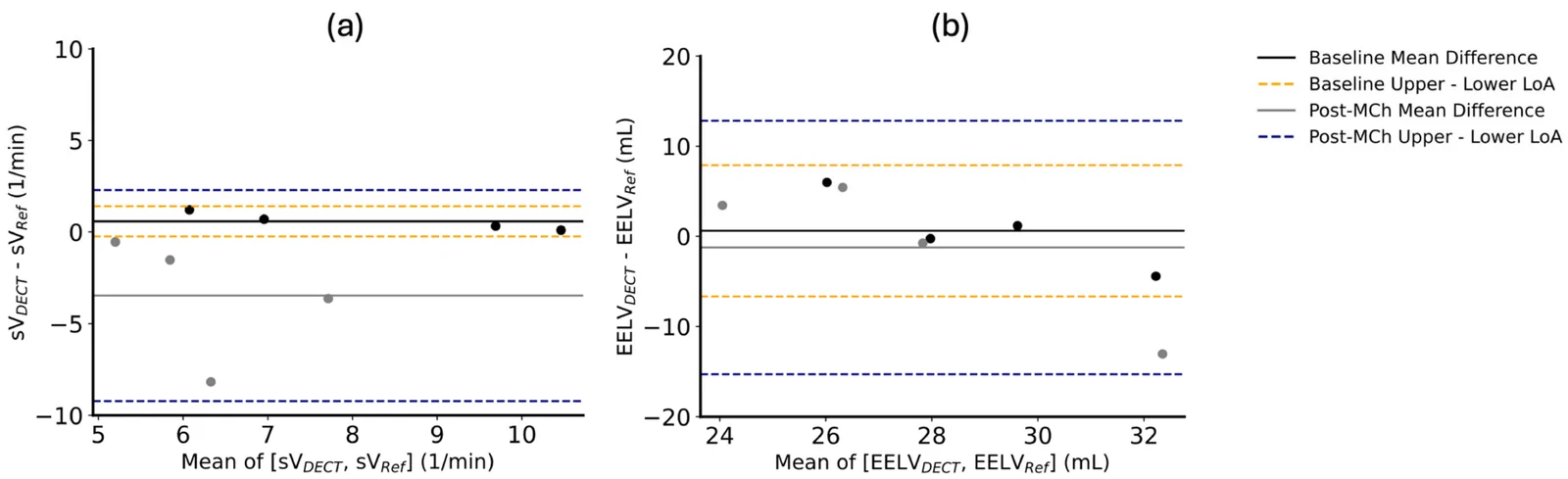
Bland–Altman analysis assessing the agreement between dual-energy CT (DECT)- and mass spectrometry-based specific ventilation (${s\dot{V}}_{\mathrm{DECT}}$, ${s\dot{V}}_{\mathrm{ms}}$ in min^−1^) (**a**) and end-expiratory lung volume (EELV_DECT_, EELV_ms_ in mL) (**b**) at baseline and after MCh administration (post-MCh). The individual results (dots) are summarized as means (solid line) with associated upper and lower limits of agreement (LoAs) computed as means ± 1.96× standard deviation (dashed line).

**Table 1:** Scan and reconstruction settings.

Parameter	
Imaging mode	Volumetric spiral shuttle
Tube voltage (kVp)	80–140 kVp switching
Tube current (mA)	200
Rotation time (s)	0.35
Collimation (mm)	80
Interscan time (s)	3.7
Pitch	0.99
Slice thickness (mm)	0.625
Matrix	512 × 512
Field-of-view (mm)	500

**Table 2:** Specific ventilation ($s\dot{V}$, min^−1^) and end-expiratory lung volume (EELV, mL) obtained from dual-energy CT (DECT) ${(s\dot{V}}_{\mathrm{DECT}}$, EELV_DECT_) and mass spectrometry ${(s\dot{V}}_{\mathrm{ms}}$, EELV_ms_) at baseline and following methacholine (MCh) administration (post-MCh). Regional ${s\dot{V}}_{\mathrm{DECT}}$ in well (${s\dot{V}}_{\mathrm{well}}$) and poorly (${s\dot{V}}_{\mathrm{poor}}$) ventilated regions is additionally reported.

$s\dot{V}$(min^−1^)	Baseline	Post-MCh
${s\dot{V}}_{\mathrm{DECT}}$	8.6 ± 1.6	3.7 ± 0.9
${s\dot{V}}_{\mathrm{poor}}$		0.2 ± 0.2
${s\dot{V}}_{\mathrm{well}}$		6.0 ± 0.4
${s\dot{V}}_{\mathrm{ms}}$	8.0 ± 2.0	6. 6 ± 0.7
EELV (mL)		
EELV_DECT_	29.3 ± 1.0	27.0 ± 1.3
EELVms	28.7 ± 4.1	28.3 ± 6.6

## Data Availability

The datasets used and/or analyzed during the current study are available from the corresponding author on reasonable request because they are subject to intellectual property restrictions and proprietary rights.

## Abbreviations

The following abbreviations are used in this manuscript:

COPD	Chronic obstructive pulmonary disease
CT	Computed tomography
CTDI_vol_	Volumetric computed tomography dose index
C_Xe_	Xenon concentration
D_air_	Air density
DECT	Dual-energy computed tomography
DLP	Dose-length product
D_l_	Lung density
D_t_	Tissue density
EELV	End-expiratory lung volume
f_air_	Gas volume fraction
FRC	Functional residual capacity
f_cor_	Tissue-volume correction factor
^68^Ga-PET	Galligas-68 positron emission tomography
LoA	Limit of agreement
MCh	Methacholine
MRI	Magnetic resonance imaging
NIST	National Institute of Standards and Technology
PCCT	Photon-counting computed tomography
SD	Standard deviation
sV	Specific ventilation
TV	Tidal volume
^99m^Tc-SPECT	Technegas-99m single-photon emission computed tomography
Xe-DECT	Xenon-enhanced dual-energy computed tomography
Xe	Xenon
V/Q	Ventilation–perfusion
Z	Atomic number
